# Supplementary figures and images for: Structure of CRL7FBXW8 reveals coupling with CUL1–RBX1/ROC1 for multi-cullin-RING E3-catalyzed ubiquitin ligation
Source: Nat Struct Mol Biol. 2022 Aug 18;29(9):854–62. doi: 10.1038/s41594-022-00815-6 (PMC9507964; doi:10.1038/s41594-022-00815-6)

Figure 4h

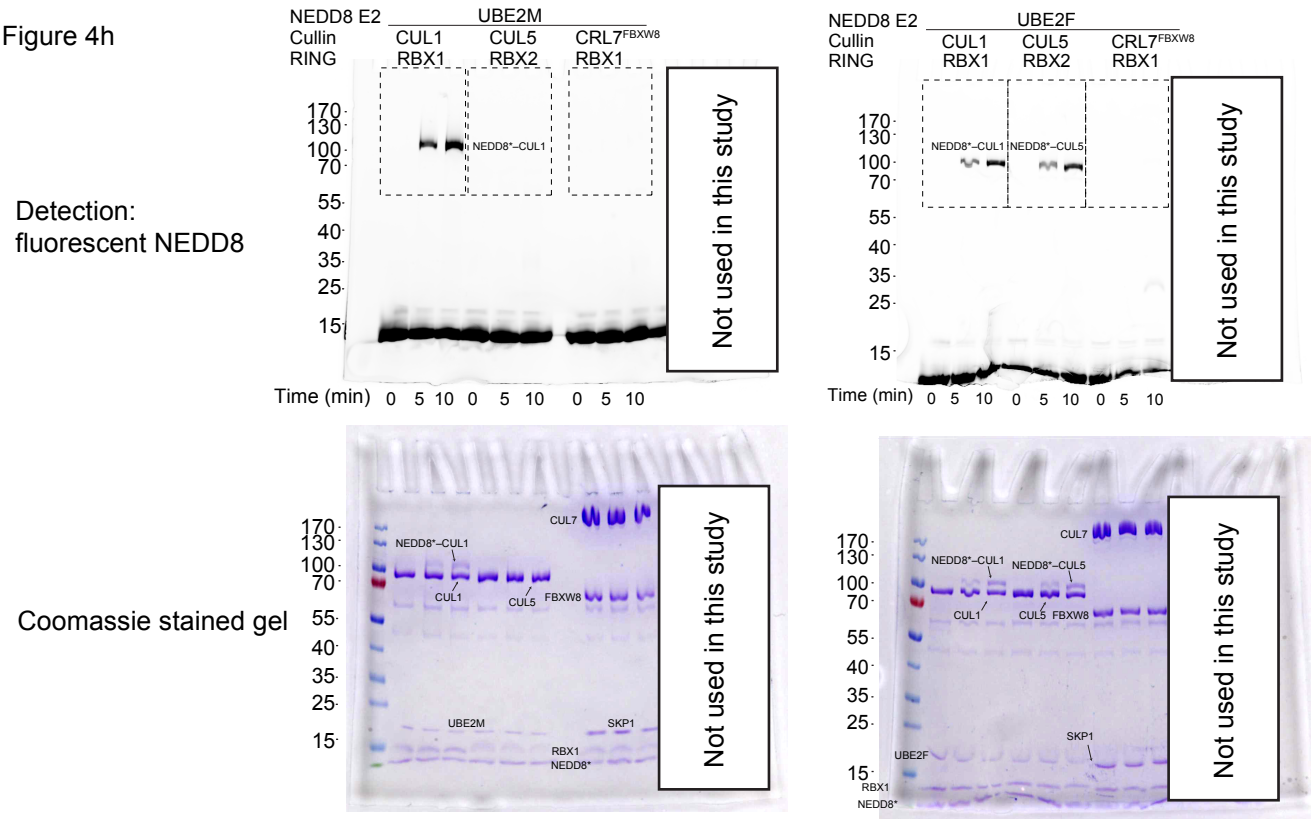

Supplement: Source Data Fig. 4 — Unprocessed gels. [file 41594_2022_815_MOESM4_ESM.pdf]

Figure 5a

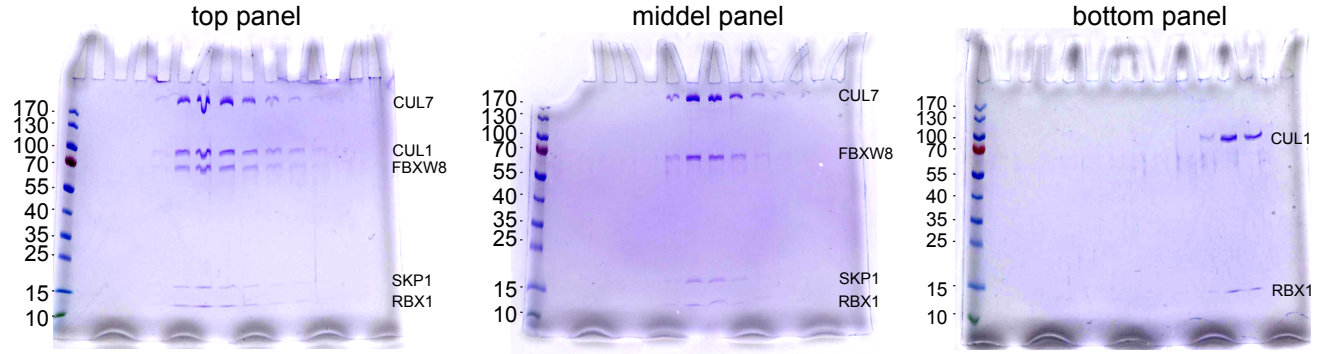

Figure 5b

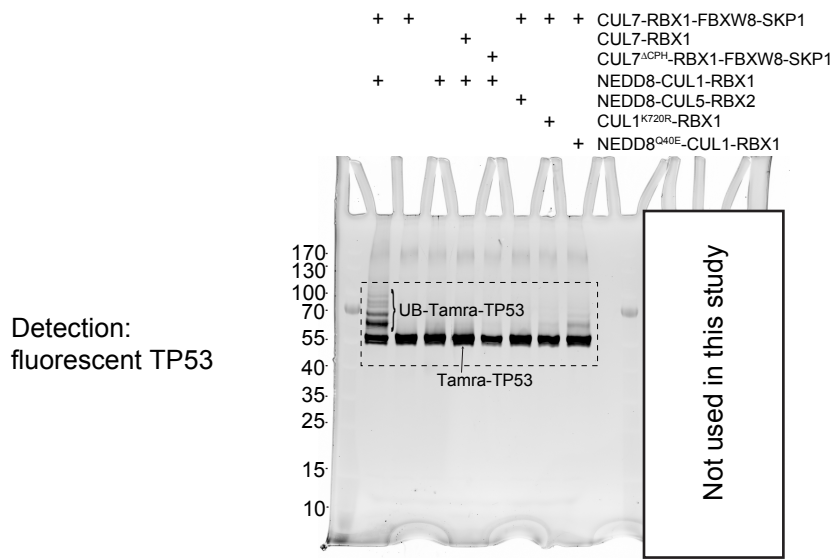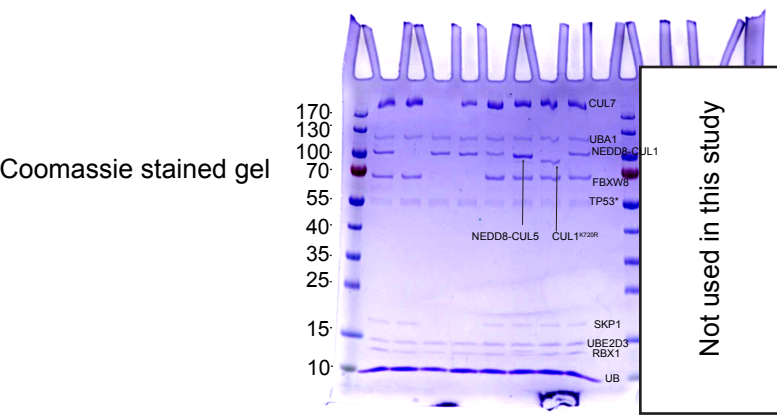

Supplement: Source Data Fig. 5 — Unprocessed gels. [file 41594_2022_815_MOESM5_ESM.pdf]

Extended Data Figure 1a

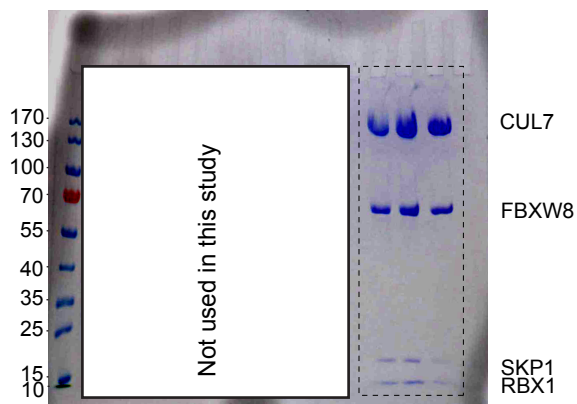

Supplement: Source Data Extended Data Fig. 1 — Unprocessed gels. [file 41594_2022_815_MOESM6_ESM.pdf]
