# Supplementary material for: Structure of CRL7FBXW8 reveals coupling with CUL1–RBX1/ROC1 for multi-cullin-RING E3-catalyzed ubiquitin ligation
Source: Nat Struct Mol Biol. 2022 Aug 18;29(9):854–62. doi: 10.1038/s41594-022-00815-6 (PMC9507964; doi:10.1038/s41594-022-00815-6)
Supplement: Source Data Extended Data Fig. 6 — Unprocessed gels. [file 41594_2022_815_MOESM7_ESM.pdf]

Extended Data Figure 6c

top panel

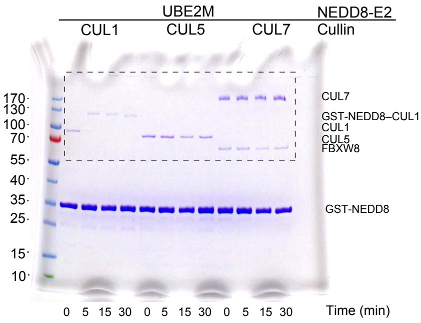

bottom panel

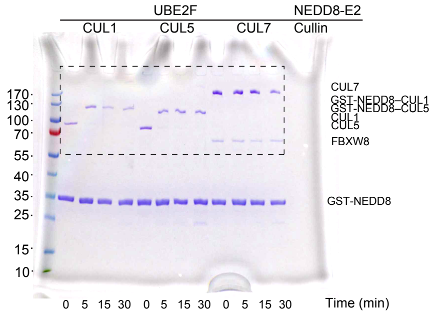

Extended Data Figure 6d

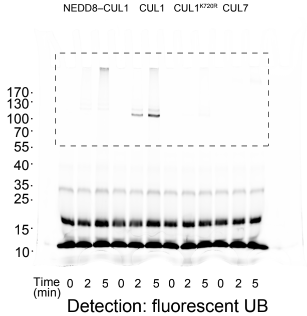

Detection: fluorescent UB

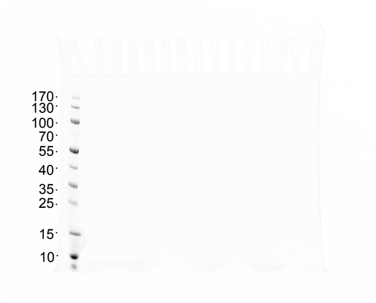

Detection: Cy5 channel (to visualize marker)

Extended Data Figure 6e

left panel

right panel

CUL7^{FBXW8} E2 screen

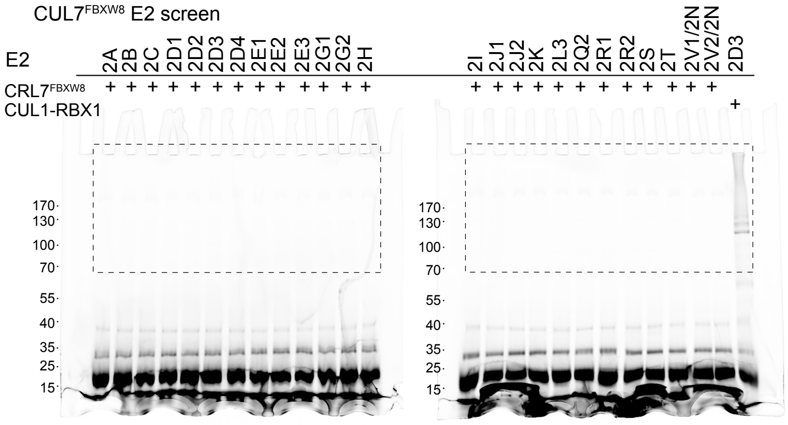

Detection: fluorescent UB

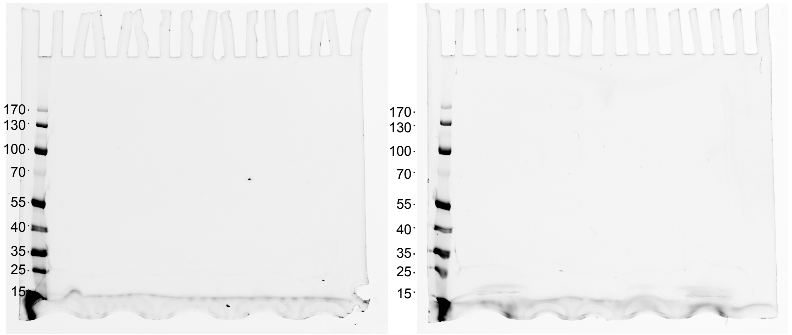

Detection: Cy5 channel (to visualize marker)

Extended Data Figure 6g

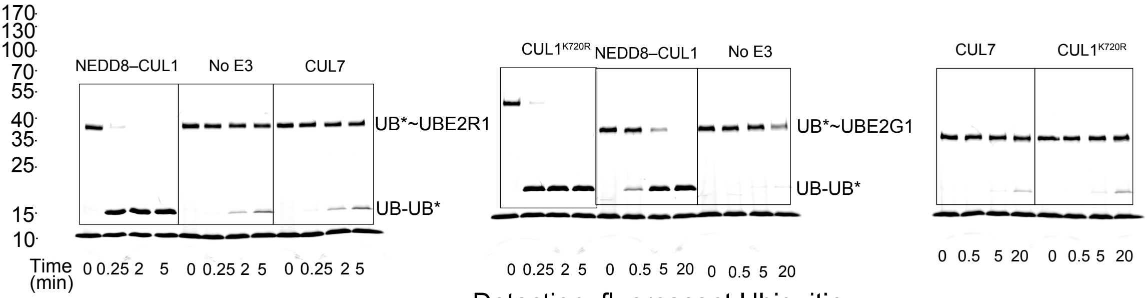

Detection: fluorescent Ubiquitin

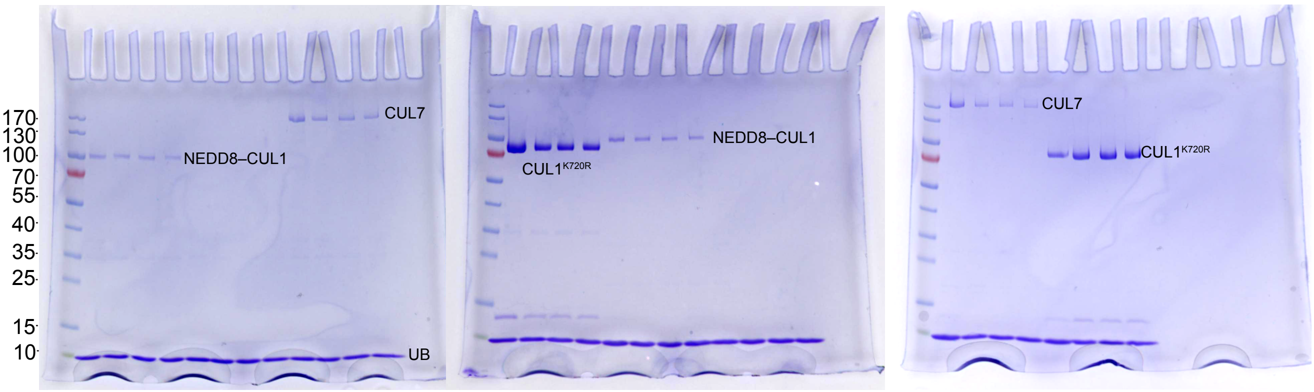

Coomassie stained gel

Loading control of proteins used in Extended Data Figure 6g

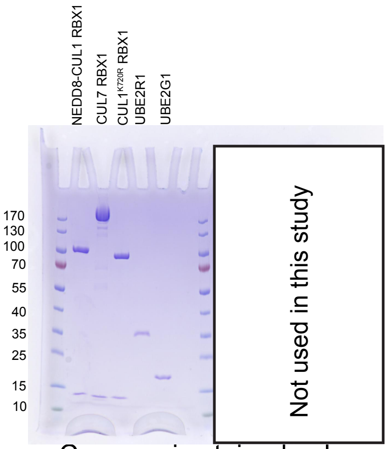

Coomassie stained gel
