# Supplementary material for: Structure of CRL7FBXW8 reveals coupling with CUL1–RBX1/ROC1 for multi-cullin-RING E3-catalyzed ubiquitin ligation
Source: Nat Struct Mol Biol. 2022 Aug 18;29(9):854–62. doi: 10.1038/s41594-022-00815-6 (PMC9507964; doi:10.1038/s41594-022-00815-6)

Extended Data Figure 7a

top panel

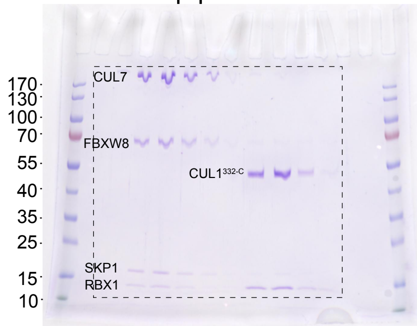

middel panel

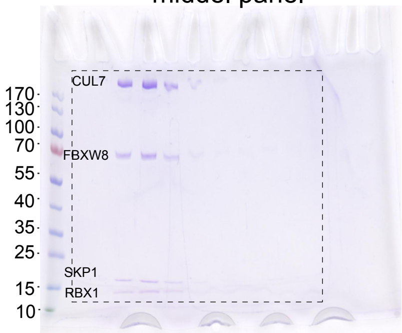

bottom panel

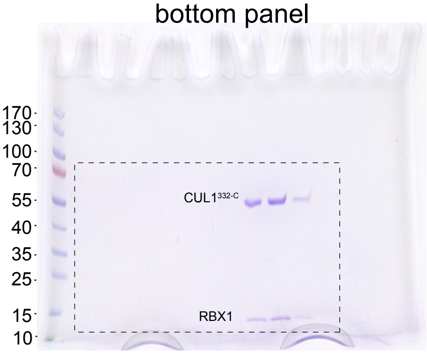

Extended Data Figure 7b

top panel
left part

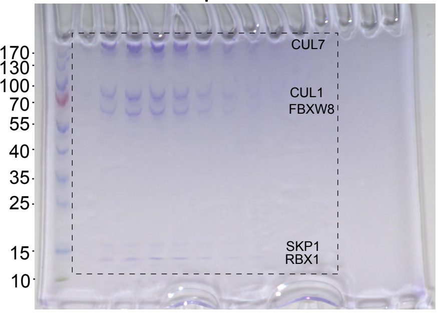

top panel
right part

bottom
panel

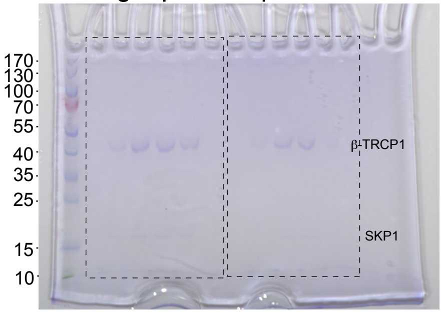

middel panel

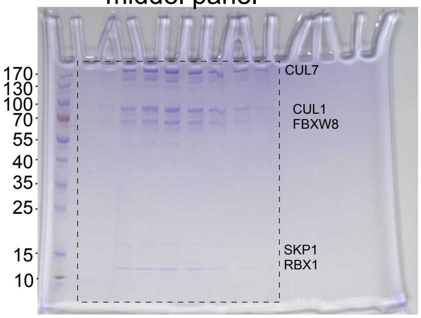

Extended Data Figure 7c

top panel

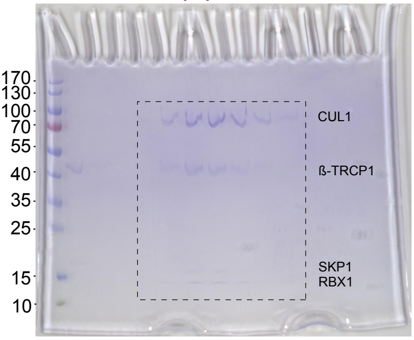

bottom panel

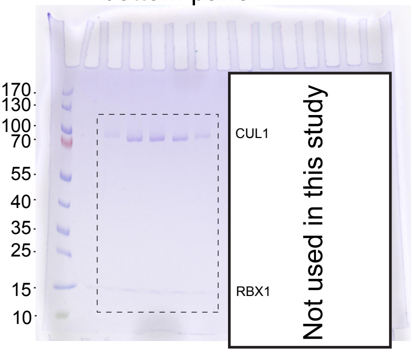

Extended Data Figure 7d

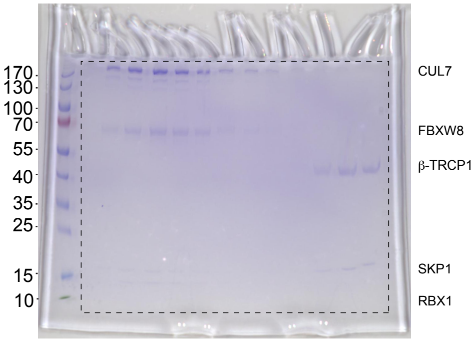

Extended Data Figure 7e

top panel

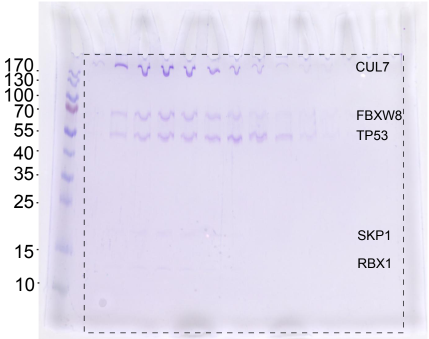

bottom panel

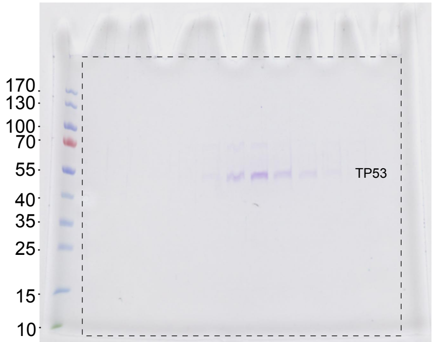

Extended Data Figure 7f

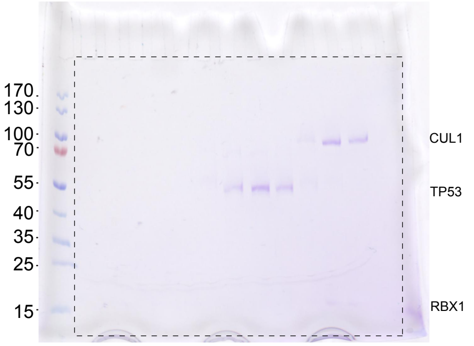

Extended Data Figure 7g

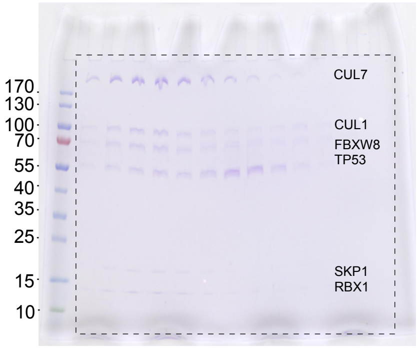

Extended Data Figure 7i

bottom panel

Extended Data Figure 7h

top panel

middel panel

bottom panel

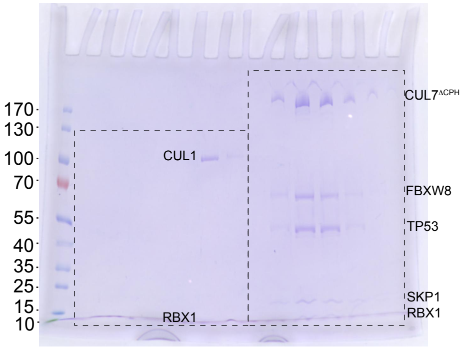

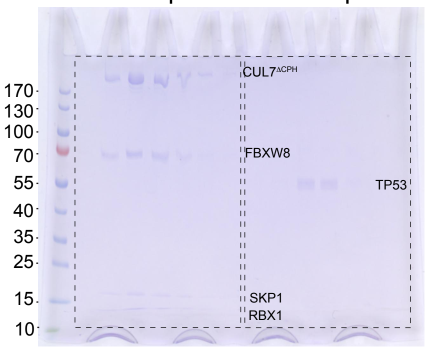

Extended Data Figure 7i

top panel

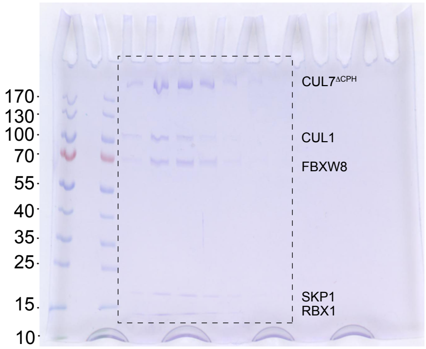

Extended Data Figure 7j

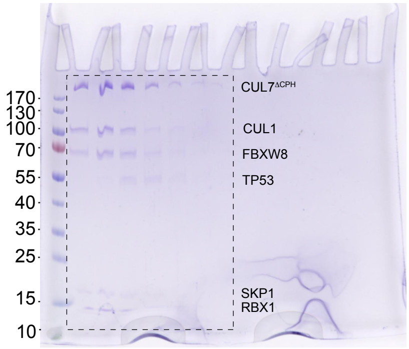

Supplement: Source Data Extended Data Fig. 7 — Unprocessed gels. [file 41594_2022_815_MOESM8_ESM.pdf]
